# The role of big trees and abundant species in driving spatial patterns of species richness in an Australian tropical rainforest

**DOI:** 10.1002/ece3.9324

**Published:** 2022-09-20

**Authors:** Helen T. Murphy, Matt G. Bradford

**Affiliations:** ^1^ CSIRO, Australian Tropical Sciences and Innovation Precinct James Cook University Townsville Queensland Australia; ^2^ CSIRO Atherton Queensland Australia

**Keywords:** accumulator, big trees, dispersal, ISAR, repeller, shade tolerance, spatial heterogeneity

## Abstract

Big trees and abundant species dominate forest structure and composition. As a result, their spatial distribution and interactions with other species and individuals may contribute disproportionately to the emergence of spatial heterogeneity in richness patterns. We tested scale‐dependent spatial patterning and species richness structures to understand the role of individual trees (big trees) and species (abundant species) in driving spatial richness patterns on a 25 ha plot in a diverse tropical forest of Australia. The individual species area relationship (ISAR) was used to assess species richness in neighborhoods ranging from 1 to 50 m radii around all big trees (≥70 cm dbh, *n* = 296) and all species with more than 100 individuals in the plot (*n* = 53). A crossed ISAR function was also used to compute species richness around big trees for trees of different size classes. Big individuals exert some spatial structuring on other big and mid‐sized trees in local neighborhoods (up to 30 m and 16 m respectively), but not on small trees. While most abundant species were neutral with respect to richness patterns, we identified consistent species‐specific signatures on spatial patterns of richness for 14 of the 53 species. Seven species consistently had higher than expected species richness in their neighborhood (species “accumulators”), and seven had lower than expected (species “repellers”) across all spatial scales. Common traits of accumulators and repeller species suggest that niche partitioning along disturbance gradients is a primary mechanism driving spatial richness patterns, which is then manifested in large‐scale spatial heterogeneity in species distributions across the plot.

## Graphical abstract

Most big trees and abundant species in a diverse tropical forest in Australia leave no strong spatial signature on tree species diversity in their surrounding neighbourhood. However, a small number of abundant species leave a consistent spatial fingerprint of higher or lower than expected tree species diversity across scales of up to 50 m radius.

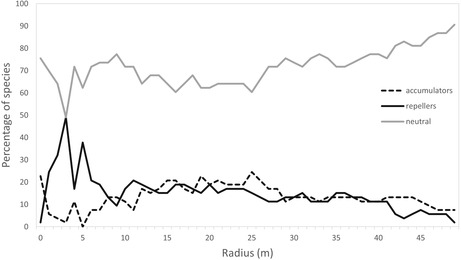

## 1 INTRODUCTION

Exceptionally high species diversity in tropical forests is thought to be maintained by multiple coexistence mechanisms that exert differential spatial structure on species (Hubbell, 1979; Wright, 2002). Niche‐based mechanisms rely on species‐specific characteristics and trade‐offs to explain how the spatial structure of species varies with environmental heterogeneity and helps to stabilize species coexistence (Brown et al., 2013). For example, trade‐offs such as high‐light growth rate versus low‐light survival can create differential spatial distributions of tree species with respect to light gradients (Poorter & Arets, 2003). Negative density dependence occurs when nearby conspecifics impair fitness through strong intra‐specific competition or host‐specific natural enemies. The Janzen–Connell hypothesis is a leading theory for how this mechanism manifests; it proposes that tree species coexist because specialized natural enemies reduce seed and seedling survival when conspecific densities are high (Connell, 1971; Janzen, 1970). A growing body of evidence supports the idea that conspecific negative density dependence is pervasive in tropical forests and a key regulating mechanism in structuring tree spatial patterns, species relative abundance, and diversity ([Comita et al., 2014; Harms et al., 2000; Johnson et al., 2012; Zhu et al., 2015] but see [Song et al., 2021]).

In many forested ecosystems, the architecture and functional ecology of certain individuals (e.g., big trees) and/or certain species (e.g. “foundation species” sensu Ellison et al. (2005)) define forest structure, their functional and physiological characteristics alter microclimates, and their biomass and chemical makeup contribute substantially to ecosystem processes (Bradford & Murphy, 2019; Lutz et al., 2013). These individuals or species may contribute disproportionately to mechanisms that drive spatial patterns in diversity because they are likely to influence the distribution and abundance of other species. For example, big trees may compete asymmetrically with small trees resulting in their respective spatial locations becoming segregated because seedlings preferentially survive and grow into understory trees where they are not suppressed by larger competitors (Lutz et al., 2012). In this case we would expect a lower density of neighbors around large trees that translates into reduced species richness at local scales. On the other hand, the Janzen–Connell hypothesis predicts that large‐sized individuals should accumulate heterospecific small‐size individuals in their local neighborhood because conspecific seedlings suffer higher mortality (Janzen, 1970), thus increasing local species richness. Ellison et al. (2005) have described tree species that are numerically abundant, large in overall size and which demonstrably influence ecological processes, as foundation species. Overall species diversity is lower in the local neighborhood of foundation species because they occupy most of the available space. Identifying these species or individuals and understanding the magnitude and scale of their influence in structuring forest communities are critical for conservation efforts and for understanding resilience and the capacity of forests to adapt to changing conditions.

The mechanisms that structure diversity operate at different scales. For example, species diversity at small scales might be influenced more strongly by competition for space with large individuals or abundant species, intra‐specific competition or inter‐specific interactions with nearby species (e.g., Hubbell et al., 2001; Lutz et al., 2013). At larger scales, species composition might be more influenced by niche differentiation along environmental gradients (Brown et al., 2013; Harms et al., 2001). The effects of particular species or individuals on diversity at multiple spatial scales have been assessed recently across a range of forest types using the individual species area relationship (ISAR) (Chanthorn et al., 2018; Punchi‐Manage et al., 2015; Tsai et al., 2015; Wiegand et al., 2007). The ISAR function can be used to compare the observed local biotic neighborhood of the individuals of focal species with that of the null model of neighborhoods of randomly selected locations (Wiegand et al., 2007). Wiegand et al. (2007) introduced the ISAR approach in their study of mega‐diverse, moist tropical forests. They found evidence that individual species leave identifiable signatures on spatial diversity at small spatial scales, but found a weak prevalence of species‐specific effects on local diversity at larger scales. Wiegand et al. (2007) explained this as the consequence of balanced multi‐specific interactions in a mild environment.

The ISAR predicts that if positive interactions with other species dominate (i.e. facilitation), the target species would be surrounded by higher than expected species richness at a particular spatial scale (i.e., being a “diversity accumulator”). For example, positive interactions between species in tropical forests may occur due to spatially contagious seed dispersal. Chanthorn et al. (2018) found that primates drive spatially contagious seed dispersal and generate species‐rich seed rain around their preferred food‐tree species at a tropical forest site in Thailand. Punchi‐Manage et al. (2015) also found higher species richness around focal species with animal‐dispersed seeds compared with those that had gravity or gyration‐dispersed seeds. In instances where negative interactions dominate (e.g., competition), there would be fewer species in an individual's neighborhood (i.e., a “diversity repeller”). However, if positive and negative interactions are weak or cancel each other out, the species behaves neutrally.

Here, we analyze scale‐dependent spatial patterning and local species richness structures to understand the role of individual trees (big trees) and species (abundant species) in driving spatial patterns of a diverse tropical forest. We used the ISAR approach to assess species richness in neighborhoods ranging from 1 to 50 m radii around all big individuals (≥70 cm dbh, *n* = 296). We further assessed, using a crossed ISAR function, whether big trees exerted differential spatial structure on species richness of small individuals (<30 cm dbh), medium‐sized individuals (30 – ≥70 cm dbh), or other big individuals. Spatial richness patterns around all species with more than 100 individuals in the plot (*n* = 53) across the 1–50 m neighborhood were also assessed. We classify species into accumulator, repeller, or neutral categories based on whether they are surrounded by more, less, or the expected number of species compared with a null model. We further broke down this analysis to determine whether repeller or accumulator patterns were consistent across all size classes of trees (big, medium, and small trees). Finally we assess common characteristics of consistent accumulator and repeller species and suggest possible mechanisms driving the observed patterns.

## 2 METHODS

**FIGURE 1:**
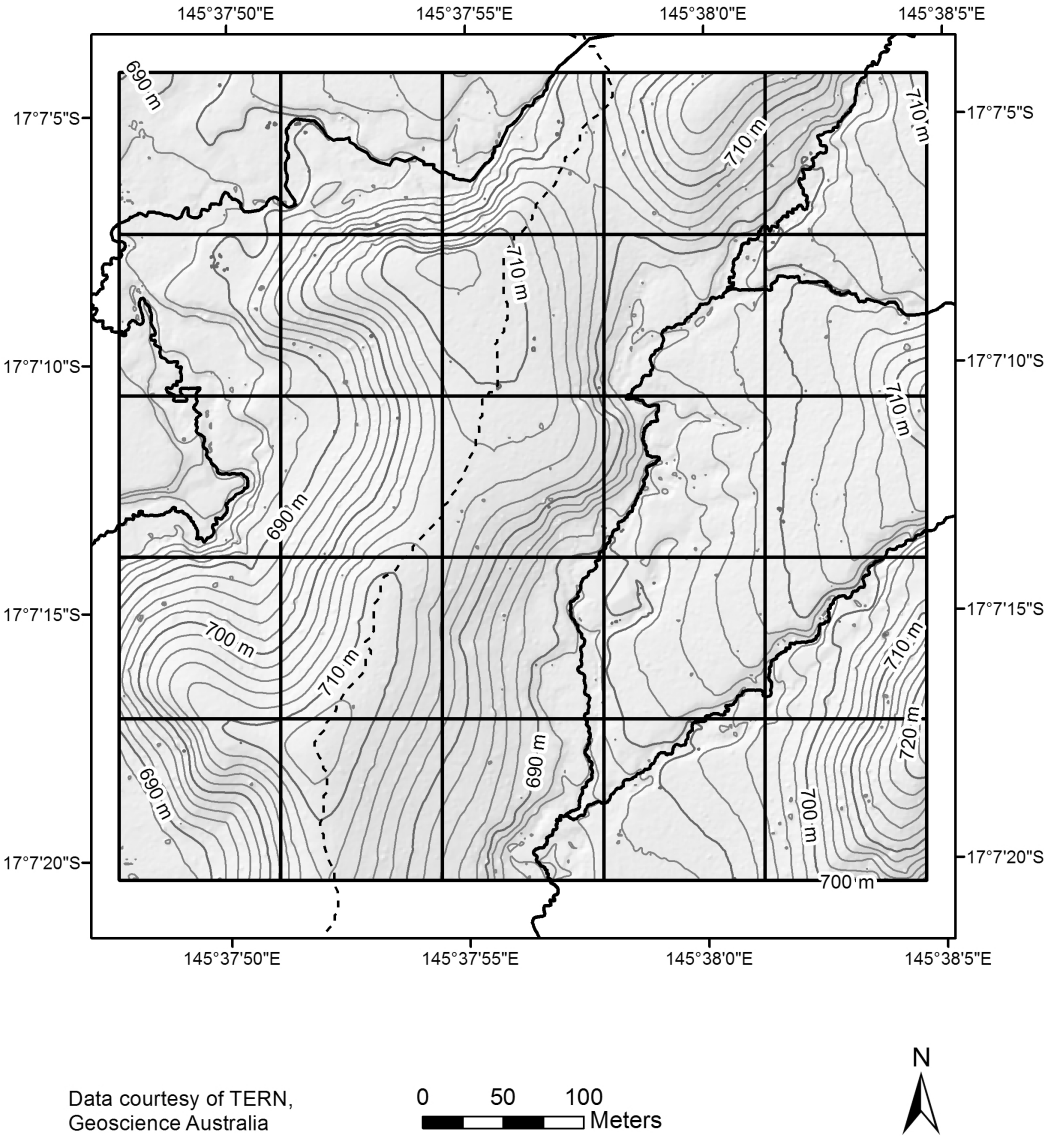
Topography of the Robson Creek plot

The Robson Creek 25 ha plot is located within the Wet Tropics World Heritage Area, North Queensland, Australia (−17.118, 145.631, Figure 1) at 680–740 m elevation. The vegetation on the plot is complex mesophyll and simple mesophyll vine forest on meta‐sediment, and soil fertility is moderately low. Canopy species attain a maximum height of 44 m although heights of 25–30 m are more common (Bradford et al., 2014). The canopy is considered uneven and no emergent stems occur. The climate of the area is seasonal with 61% of annual rainfall occurring between January and March. Mean annual rainfall is approximately 1600 mm (1921–2020).

Structurally and floristically defining features of Australian wet tropical rainforests are frequent disturbance by tropical cyclones (hurricanes/typhoons) and affinities with both Indo‐Malayan and Gondwanan taxa (Metcalfe & Ford, 2009). Cyclones are important structuring events of rainforests in the Wet Tropics of Australia with historical data suggesting that a severe cyclone will cross any particular point on the coast at least once every 75 years (Turton & Stork, 2009). Severe Cyclone Larry caused moderate structural damage to the plot in 2006. Long‐term monitoring at an adjacent plot revealed mortality of 74 trees ha^−1^ >10 cm dbh (Metcalfe et al., 2008; Murphy et al., 2013). In addition, as is the case with most accessible areas of rainforest in Australia, the plot was selectively logged, with the last logging activity occurring between 1960 and 1969 (Bradford et al., 2014). Logging records for the plot have not been retained; however, extraction rates in a nearby area were ~ 6.6 trees ha^−1^ with incidental damage caused by logging activities causing up to 22% canopy loss (Crome et al., 1992).

All stems on the plot with a diameter at breast height (dbh) ≥10 cm (trees, lianas, ferns, strangler figs) were identified to species, mapped, and the height and dbh measured (Bradford, 2018). Stems were mapped to an accuracy of ±0.5 m. The dbh was measured according to protocols outlined in Condit (1998) with one variation: for species known to exhibit buttressing on larger specimens, the point of measurement was preemptively elevated above the predicted buttressing influence. The stem census took place between December 2009 and November 2012. The plot comprises 23, 416 individuals (≥10 cm dbh) of 207 species (Table 1).

**TABLE 1:** Number and proportion (in brackets) of individuals and species at Robson Creek for all trees and for small, mid and big trees

	Individuals	Species
All trees	23,416	207
Small (≥10 < 30 cm dbh)	19,619 (83.7%)	201 (97%)
Mid‐size (≥30 <70 cm dbh)	3501 (14.9%)	126 (61%)
Big (≥70 cm dbh)	296 (1.2%)	42 (20%)

The ISAR (individual species area relationship) function computes the mean species richness within distance *r* of the individuals of a given focal species/group *f*. Comparison of the observed ISAR function with that of multiple realizations of a suitable null model (where the focal species locations are compared with random locations in the plot) reveals whether a focal species is surrounded by local species assemblages of lower or higher than expected species richness (Wiegand et al., 2007). We compared observed fits of the ISAR function against the expectation under an inhomogeneous Poisson null model, which is implemented using a non‐parametric Gaussian kernel estimation of the spatially varying intensity function of the focal species. Thus, the null model was based on an intensity surface of the focal group, which takes account of “first‐order effects” in the spatial distribution of individuals related to unmeasured large scale environmental heterogeneity and controls for the effects of habitat association. It does this by displacing the known locations of trees in the focal group within a neighborhood of a given bandwidth, while fixing the locations of other individuals. We chose a maximum bandwidth of 50 m, which covers the range of scales where both local interactions (i.e., competition) and niche partitioning (e.g., in light gaps) predominantly occur, and which is consistent with other analyses in tropical forests for comparison (Tsai et al., 2015; Wiegand et al., 2007).

Species richness of small (≥10 – 30 cm dbh), medium (≥30 – <70 cm dbh), and big trees (≥70 cm dbh) (Table 1) was calculated within a 50 m radius incorporating a 10 m edge buffer using the ISAR function. Species density across the plot was also calculated for small, medium, and big trees.

We used the ISAR function to compute local species richness around (a) all big trees (*n* = 296) and (b) all species with >100 individuals in the plot (*n* = 53). A crossed ISAR function was used to compute species richness around big trees for (a) all small trees, (b) mid‐sized trees, and (c) other big trees (Table 1). The ISAR function was computed for neighborhoods of 1–50 m radius (*r*) at 1 m intervals with no edge correction (Wiegand & Moloney, 2014). We computed Monte Carlo simulation envelopes for each focal species/group based on 199 simulations of the fitted inhomogeneous null model. We determined the fifth highest and fifth lowest values of the ISAR(*r*) to generate confidence envelopes. If the observed ISAR(*r*) was larger than the 5th highest ISAR(r) of the 199 simulations of the null model than the focal species/group was considered to have accumulated higher than expected species diversity (i.e., accumulator). If the observed ISAR(r) was lower than the fifth lowest ISAR(r) of the 199 simulations, the focal species/group was considered to have lower than expected species richness (i.e., repeller).

For species that were considered accumulators or repellers based on the ISAR test, we then used a maximum absolute deviation (MAD) goodness of fit test to assess the significance of deviations from the null model. This test reduces type 1 error inflation due to multiple simulations (Loosmore & Ford, 2006; Wiegand et al., 2016). We tested the significance of deviations over 10 m increments from 1–50 m (i.e., 1–10, 11–20, 21–30, 31–40, 41–50 m). The observed ISAR(*r*) and each of the 199 simulated ISARs of the null model are reduced to a single summary statistic that represents the total squared deviation between the observed and theoretical ISAR at each increment. The rank of the summary statistic of the observed ISAR was used for the goodness of fit test. Thus, a significant departure from the null model occurred for an α of 0.05 when the rank of the observed summary statistic was great than 190 (Nguyen et al., 2018; Wiegand et al., 2007).

All analyses were done using RStudio 2022.02.3 + 492 (RStudio Team, 2019) with R 4.2.0 (R Core Team, 2022), using the packages *idar v1.1* (Chacón‐Labella et al., 2016; Espinosa et al., 2016) and *spatstat v1.62–2* (Baddeley et al., 2015).

## 3 RESULTS

**FIGURE 2:**
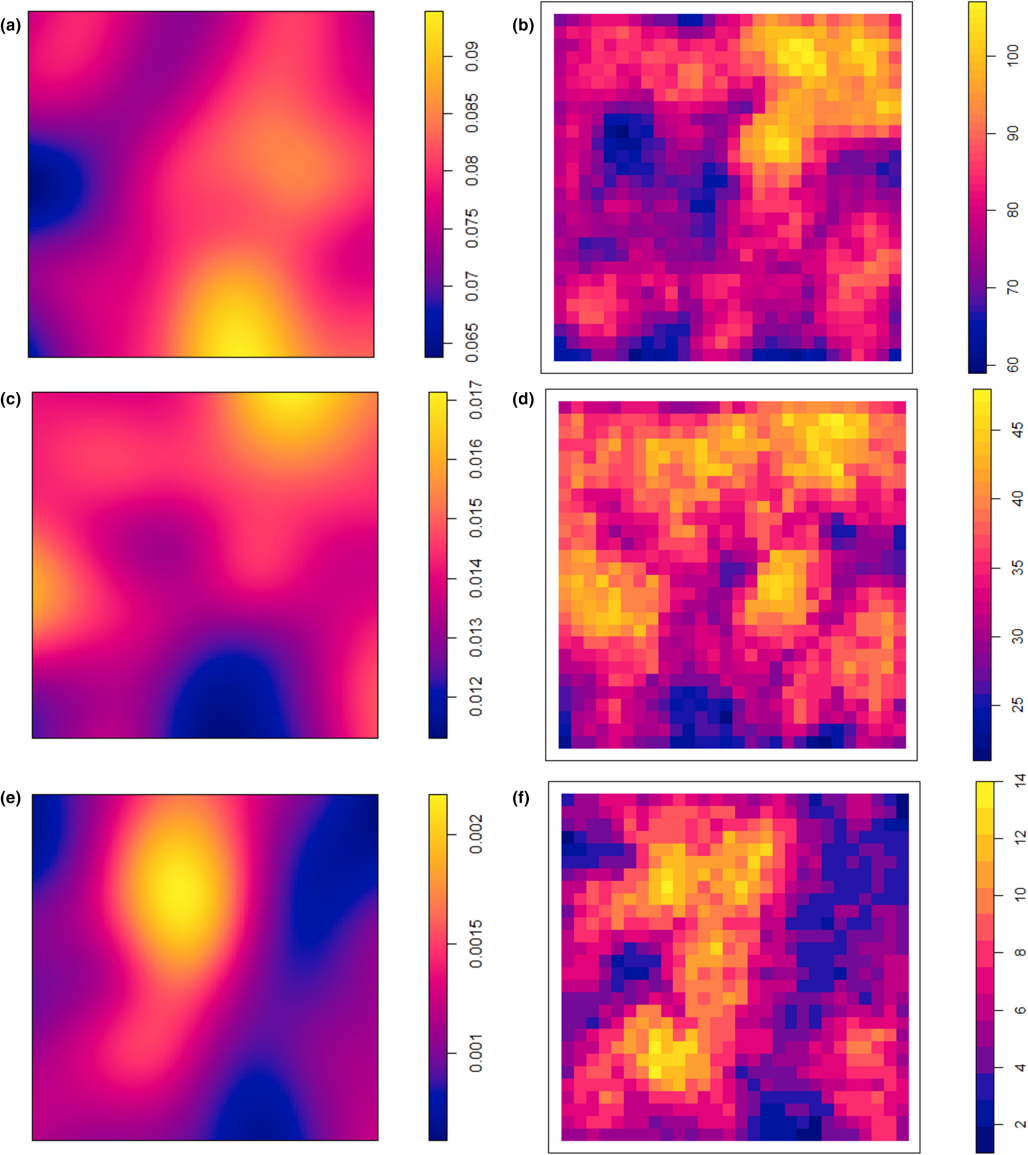
Density of (a) small, (c) medium, and (e) big trees and spatial variability of local species richness at a radius of 50 m for (b) small, (d) medium, and (f) big trees with a 10 m edge buffer

Peaks of species richness and density for small trees occur in the flatter, lower‐lying areas of the plot in the east and south‐east; however, the areas of highest species richness and density occur in different locations on the plot, in the north‐east and south‐east, respectively (Figure 2a,b). Species richness of small trees ranges from 60 to 110 species at a 50 m radius across the plot (with a 10 m edge buffer) (Figure 2b). Species richness of medium‐sized tree ranges from 20 to 50 species at a 50 m radius (Figure 2d) and is relatively evenly distributed across the plot, but species density (Figure 2c) peaks in the north east and central west of the plot. Species richness and density of big trees are highest in the central areas of the plot (Figure 2e,f) between two drainage lines (Figure 1b). For big trees, species richness ranges from 1 to 14 species at a 50 m radius (Figure 2f).

**FIGURE 3:**
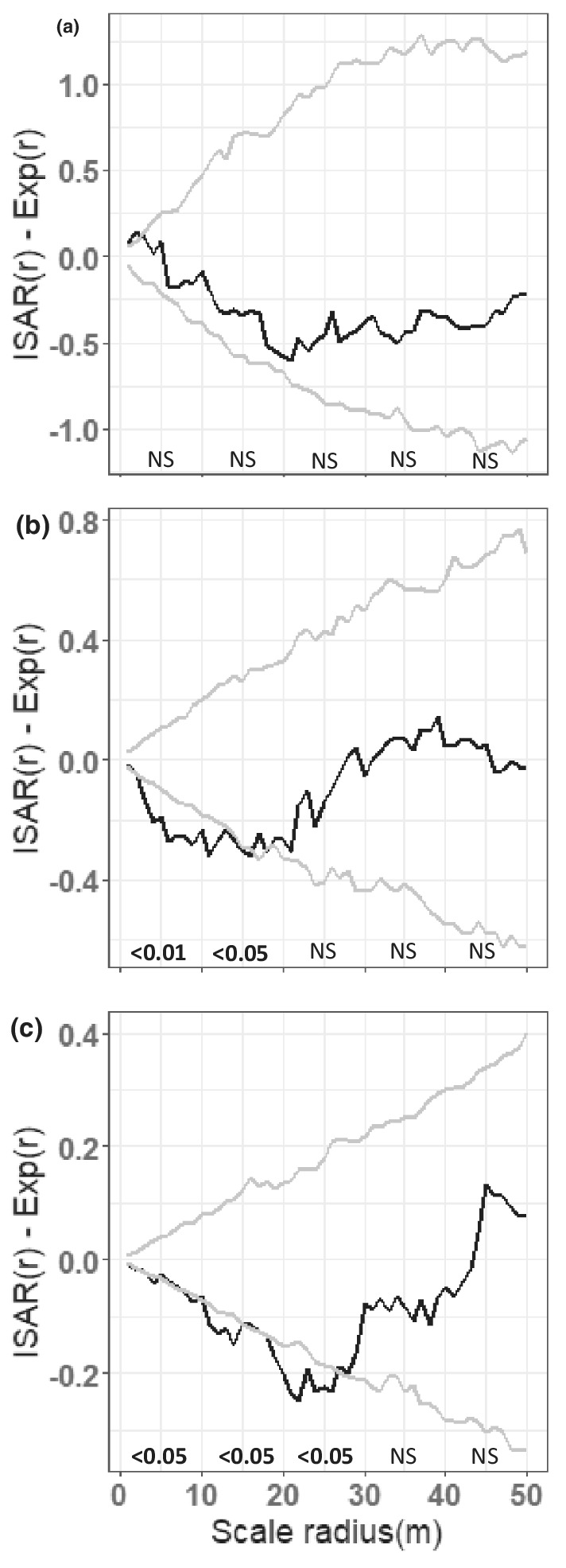
Result of crossed ISAR analysis for (a) small trees (b) mid‐size trees, and (c) big trees around all big trees. The simulation envelopes (gray lines) represent the fifth lowest and highest values of the ISAR(r) – Exp(r) of the 199 simulations of the null model. Text above the x‐axis indicates the result of the goodness of fit (MAD) test, i.e., significant departure from the null model at the <0.05 or <0.01 level or not a significant departure from the null model (NS)

The individual species area relationship analysis reveals that the species richness of small trees is neutral with respect to the distribution of big trees; however, the species richness of mid‐sized trees is lower than expected at distances up to about 16 m around big trees (Figure 3a,b). Big trees repel other big trees up to 27 m (Figure 3c).

**FIGURE 4:**
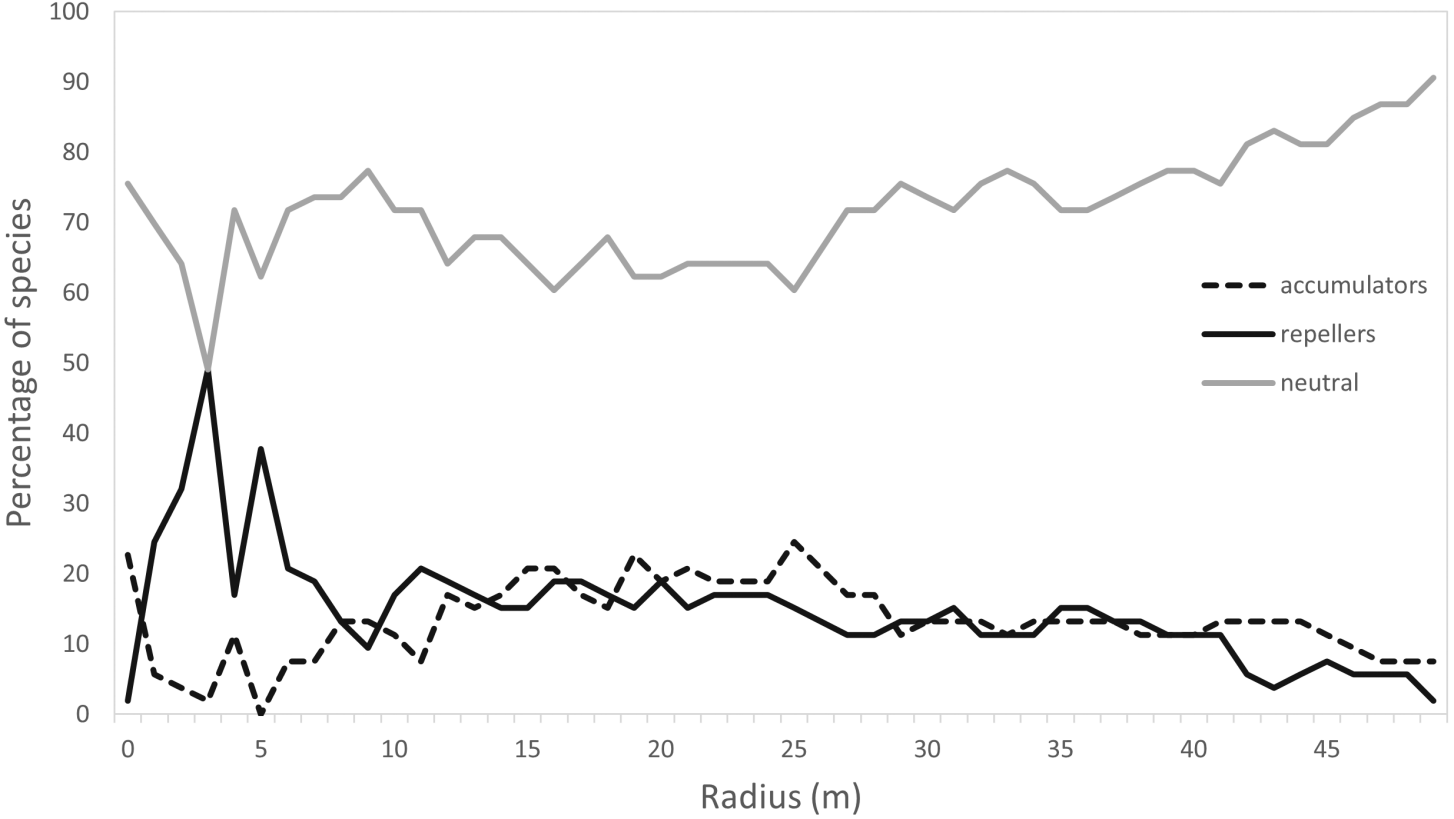
Proportion of accumulator, repeller, and neutral species among the 53 most abundant species (species with >100 individuals) at radii of 1–50 m

The proportion of accumulators (species with higher than expected species richness in a given neighborhood), repellers (lower than expected species richness), and neutral species among the 53 species with >100 individuals is shown in Figure 4. At all scales most species are neutral with respect to structuring species diversity. At the closest radii (1 m) 22% of species (*n* = 12) accumulate more species than expected with only one individual repelling. However, given tree location accuracy is ~0.5 m, this result should be regarded with some caution. At radii between 2 and 8 m, the number of repellers is greater than the number of accumulators. At 4 m nearly 50% of the 53 species (*n* = 26) have lower than expected species richness (repellers) compared with only one species that has higher than expected species richness. Beyond a 10 m radius, the proportions of accumulators and repellers are relatively steady (15%–20%) with the proportion of accumulators mostly slightly higher than repellers. The proportion of neutral species gradually increases beyond a 25 m radius.

**FIGURE A1:**
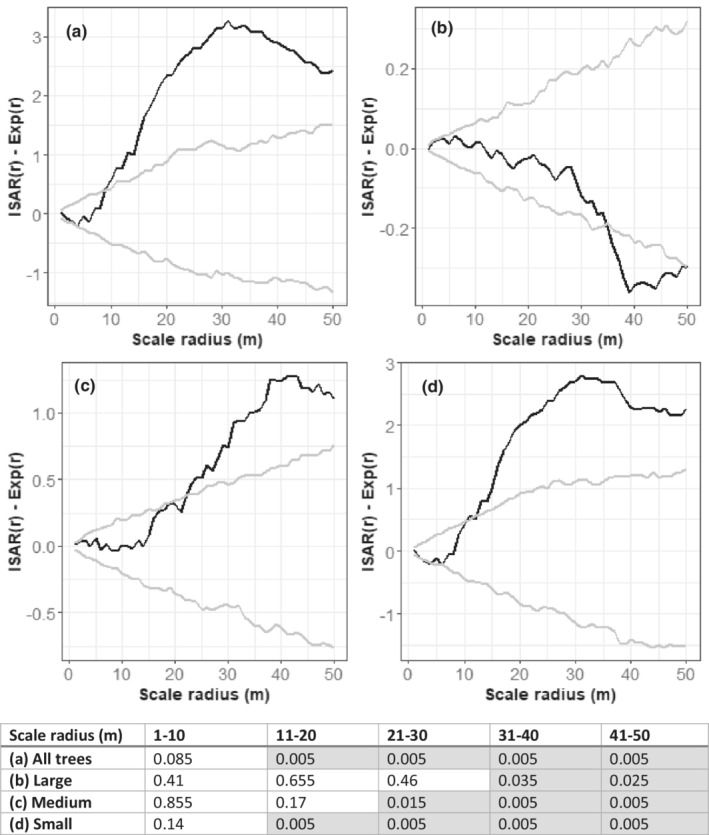
Results of ISAR analyses for *Apodytes brachystylis* (accumulator). Black lines show ISAR observed – ISAR expected, which represents the observed ISAR function minus the expectation under a heterogenous Poisson null model. Gray lines represent the fifth highest and fifth lowest values of the 199 simulations of the null model. ISAR results are shown for (a) all individuals, (b) large individuals (≥70 cm dbh), (c) mid‐sized individuals only (≥30 and <70 cm dbh), and (d) small individuals only (<30 cm dbh). The table gives the significance level (*p*) of the MAD goodness of fit test at increments of 10 m. Shaded cells highlight a result indicating a significant departure of the observed ISAR(r) from the null model ISAR at that particular increment

**FIGURE A2:**
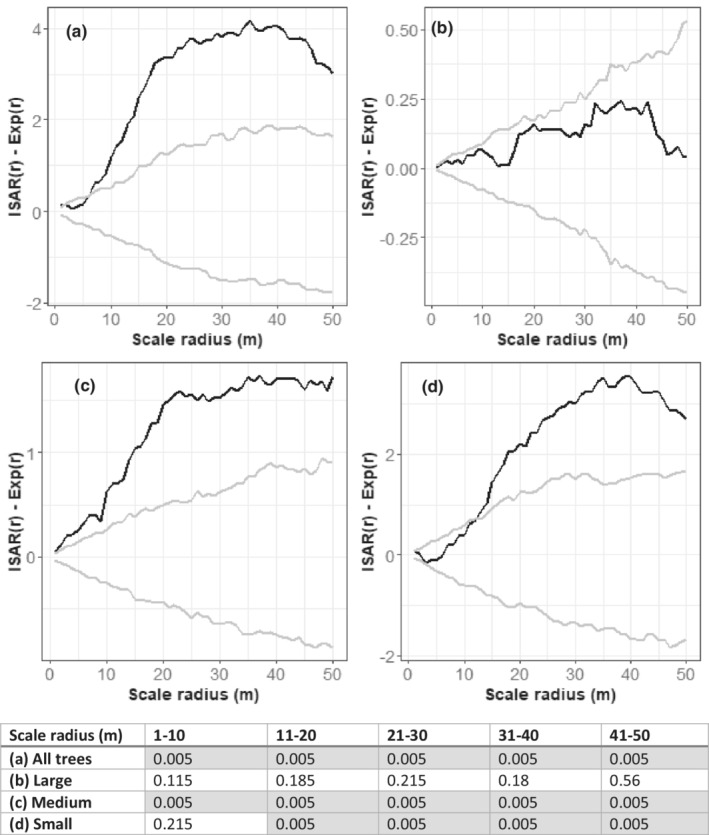
Results of ISAR analyses for *Citronella smythii* (accumulator). Black lines show ISAR observed – ISAR expected, which represents the observed ISAR function minus the expectation under a heterogenous Poisson null model. Gray lines represent the fifth highest and fifth lowest values of the 199 simulations of the null model. ISAR results are shown for (a) all individuals, (b) large individuals (≥70 cm dbh), (c) mid‐sized individuals only (≥30 and <70 cm dbh), and (d) small individuals only (<30 cm dbh). The table below gives the significance level (*p*) of the MAD goodness of fit test at increments of 10 m. Shaded cells highlight a result indicating a significant departure of the observed ISAR(r) from the null model ISAR at that particular increment

**FIGURE A3:**
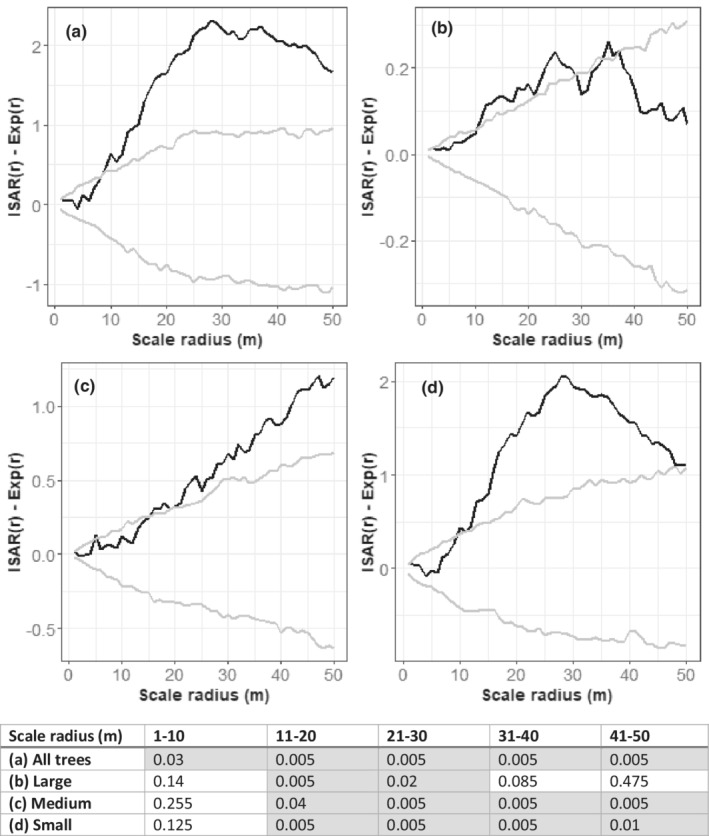
Results of ISAR analyses for *Franciscodendron laurifolium* (accumulator). Black lines show ISAR observed – ISAR expected, which represents the observed ISAR function minus the expectation under a heterogenous Poisson null model. Gray lines represent the fifth highest and fifth lowest values of the 199 simulations of the null model. ISAR results are shown for (a) all individuals, (b) large individuals (≥70 cm dbh), (c) mid‐sized individuals only (≥30 and <70 cm dbh), and (d) small individuals only (<30 cm dbh). The table below gives the significance level (p) of the MAD goodness of fit test at increments of 10 m. Shaded cells highlight a result indicating a significant departure of the observed ISAR(r) from the null model ISAR at that particular increment

**FIGURE A4:**
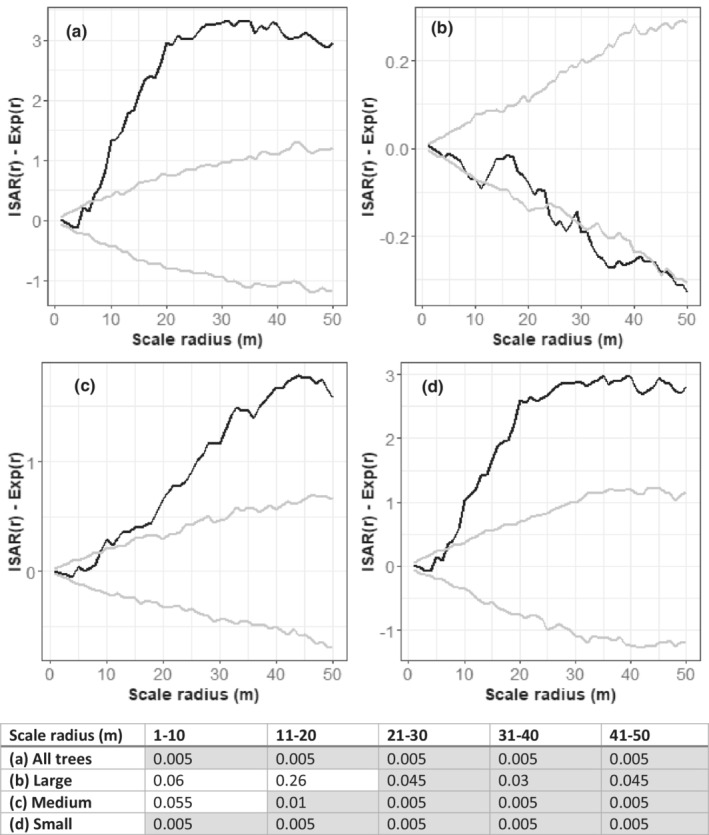
Results of ISAR analyses for *Gillbeea adenopetala* (accumulator). Black lines show ISAR observed – ISAR expected, which represents the observed ISAR function minus the expectation under a heterogenous Poisson null model. Gray lines represent the fifth highest and fifth lowest values of the 199 simulations of the null model. ISAR results are shown for (a) all individuals, (b) large individuals (≥70 cm dbh), (c) mid‐sized individuals only (≥30 and <70 cm dbh), and (d) small individuals only (< 30 cm dbh). The table below gives the significance level (p) of the MAD goodness of fit test at increments of 10 m. Shaded cells highlight a result indicating a significant departure of the observed ISAR(r) from the null model ISAR at that particular increment

**FIGURE A5:**
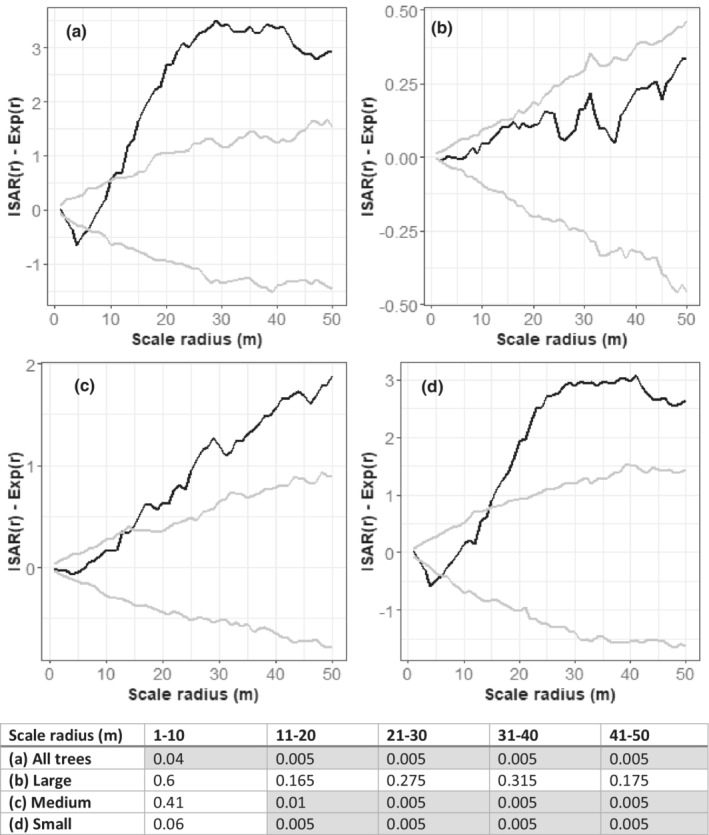
Results of ISAR analyses for *Levieria acuminata* (accumulator). Black lines show ISAR observed – ISAR expected, which represents the observed ISAR function minus the expectation under a heterogenous Poisson null model. Gray lines represent the fifth highest and fifth lowest values of the 199 simulations of the null model. ISAR results are shown for (a) all individuals, (b) large individuals (≥70 cm dbh), (c) mid‐sized individuals only (≥30 and <70 cm dbh), and (d) small individuals only (<30 cm dbh). The table below gives the significance level (*p*) of the MAD goodness of fit test at increments of 10 m. Shaded cells highlight a result indicating a significant departure of the observed ISAR(r) from the null model ISAR at that particular increment

**FIGURE A6:**
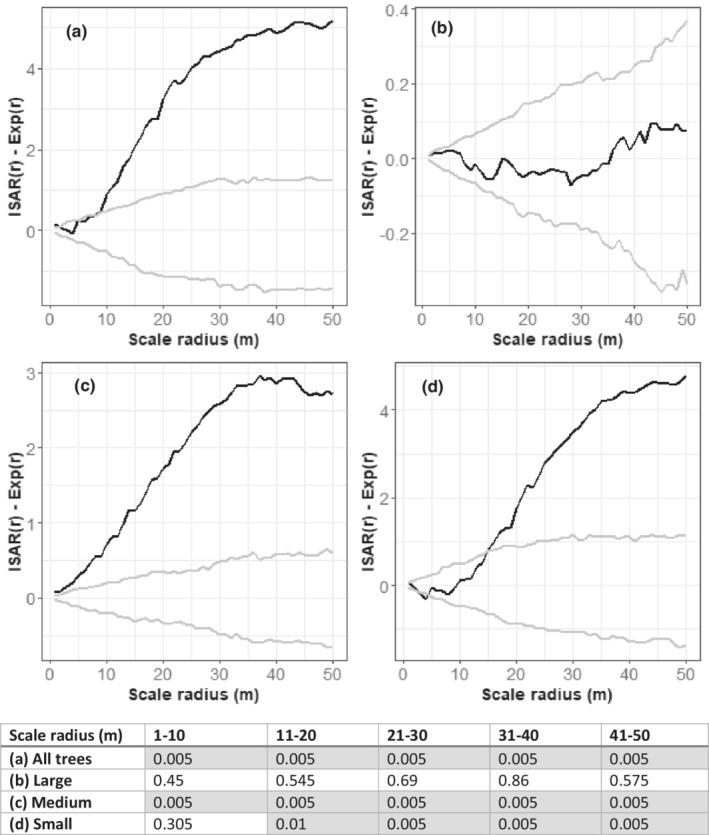
Results of ISAR analyses for *Medicosma fareana* (accumulator). Black lines show ISAR observed – ISAR expected, which represents the observed ISAR function minus the expectation under a heterogenous Poisson null model. Gray lines represent the fifth highest and fifth lowest values of the 199 simulations of the null model. ISAR results are shown for (a) all individuals, (b) large individuals (≥70 cm dbh), (c) mid‐sized individuals only (≥30 and <70 cm dbh), and (d) small individuals only (< 30 cm dbh). The table below gives the significance level (*p*) of the MAD goodness of fit test at increments of 10 m. Shaded cells highlight a result indicating a significant departure of the observed ISAR(r) from the null model ISAR at that particular increment

**FIGURE A7:**
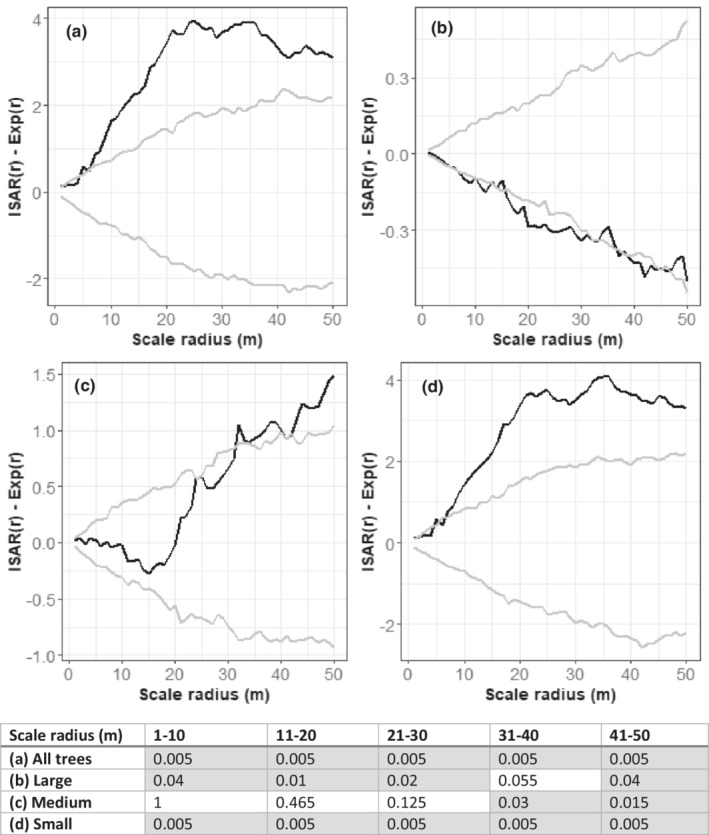
Results of ISAR analyses for *Symplocos paucistaminea* (accumulator). Black lines show ISAR observed – ISAR expected, which represents the observed ISAR function minus the expectation under a heterogenous Poisson null model. Gray lines represent the fifth highest and fifth lowest values of the 199 simulations of the null model. ISAR results are shown for (a) all individuals, (b) large individuals (≥70 cm dbh), (c) mid‐sized individuals only (≥30 and <70 cm dbh), and (d) small individuals only (<30 cm dbh). The table below gives the significance level (*p*) of the MAD goodness of fit test at increments of 10 m. Shaded cells highlight a result indicating a significant departure of the observed ISAR(r) from the null model ISAR at that particular increment

**FIGURE A8:**
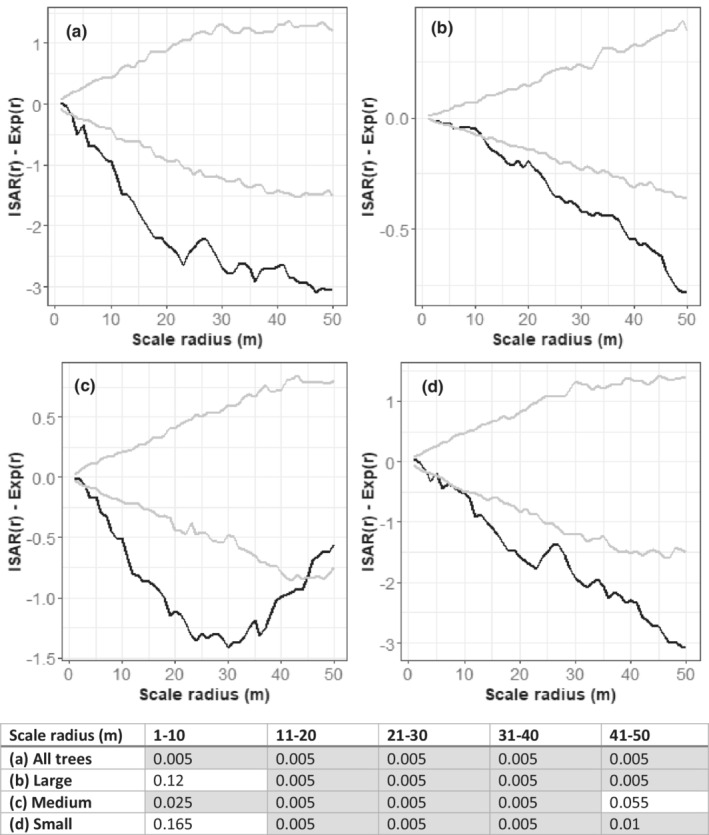
Results of ISAR analyses for *Acronychia vestita* (repeller). Black lines show ISAR observed – ISAR expected, which represents the observed ISAR function minus the expectation under a heterogenous Poisson null model. Gray lines represent the fifth highest and fifth lowest values of the 199 simulations of the null model. ISAR results are shown for (a) all individuals, (b) large individuals (≥70 cm dbh), (c) mid‐sized individuals only (≥30 and <70 cm dbh), and (d) small individuals only (<30 cm dbh). The table below gives the significance level (*p*) of the MAD goodness of fit test at increments of 10 m. Shaded cells highlight a result indicating a significant departure of the observed ISAR(r) from the null model ISAR at that particular increment

**FIGURE A9:**
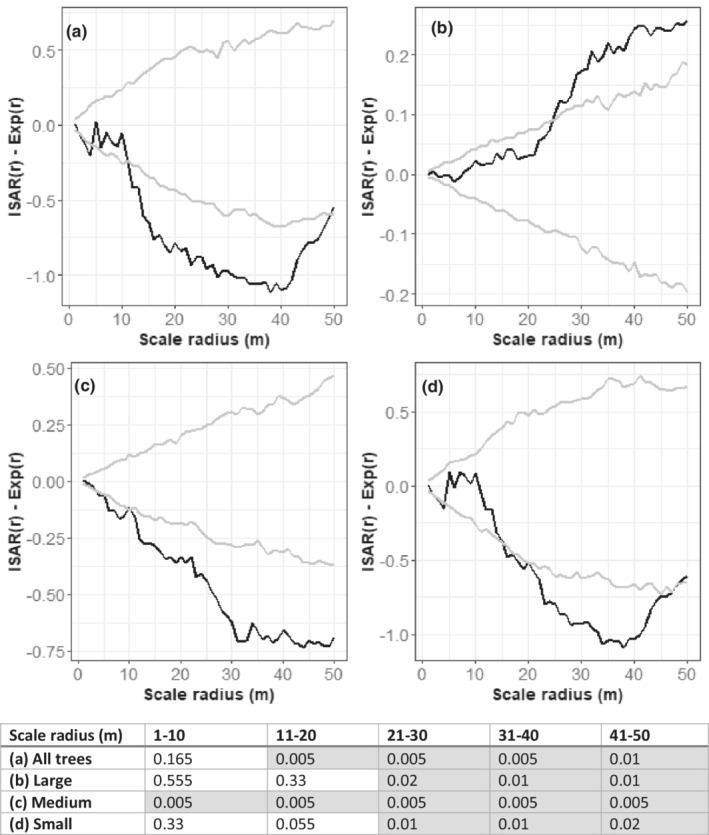
Results of ISAR analyses for *Alphitonia whitei* (repeller). Black lines show ISAR observed – ISAR expected, which represents the observed ISAR function minus the expectation under a heterogenous Poisson null model. Gray lines represent the fifth highest and fifth lowest values of the 199 simulations of the null model. ISAR results are shown for (a) all individuals, (b) large individuals (≥70 cm dbh), (c) mid‐sized individuals only (≥30 and <70 cm dbh), and (d) small individuals only (< 30 cm dbh). The table below gives the significance level (*p*) of the MAD goodness of fit test at increments of 10 m. Shaded cells highlight a result indicating a significant departure of the observed ISAR(r) from the null model ISAR at that particular increment

**FIGURE A10:**
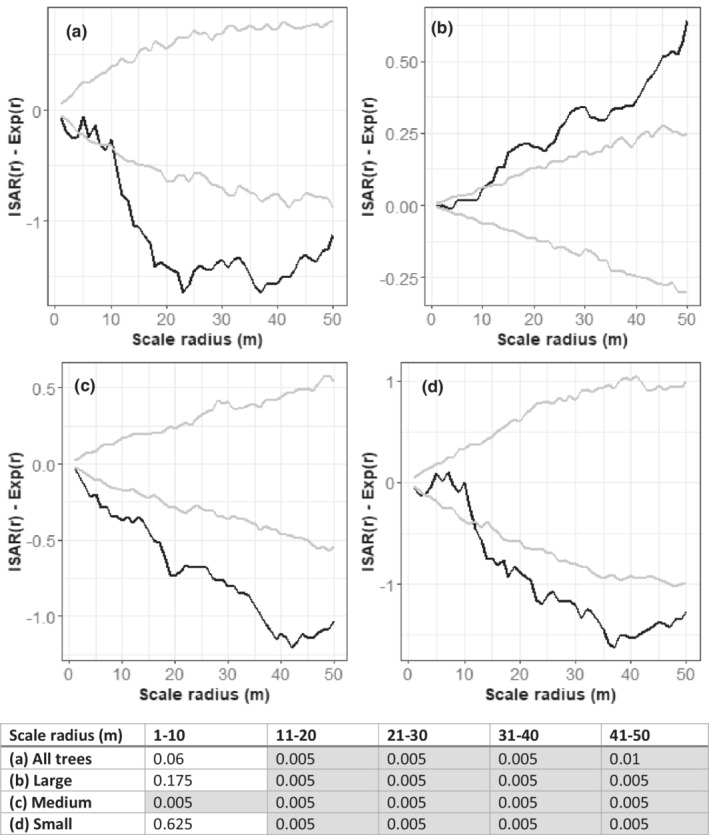
Results of ISAR analyses for *Alstonia muelleriana* (repeller). Black lines show ISAR observed – ISAR expected, which represents the observed ISAR function minus the expectation under a heterogenous Poisson null model. Gray lines represent the fifth highest and fifth lowest values of the 199 simulations of the null model. ISAR results are shown for (a) all individuals, (b) large individuals (≥70 cm dbh), (c) mid‐sized individuals only (≥30 and <70 cm dbh), and (d) small individuals only (<30 cm dbh). The table below gives the significance level (*p*) of the MAD goodness of fit test at increments of 10 m. Shaded cells highlight a result indicating a significant departure of the observed ISAR(r) from the null model ISAR at that particular increment

**FIGURE A11:**
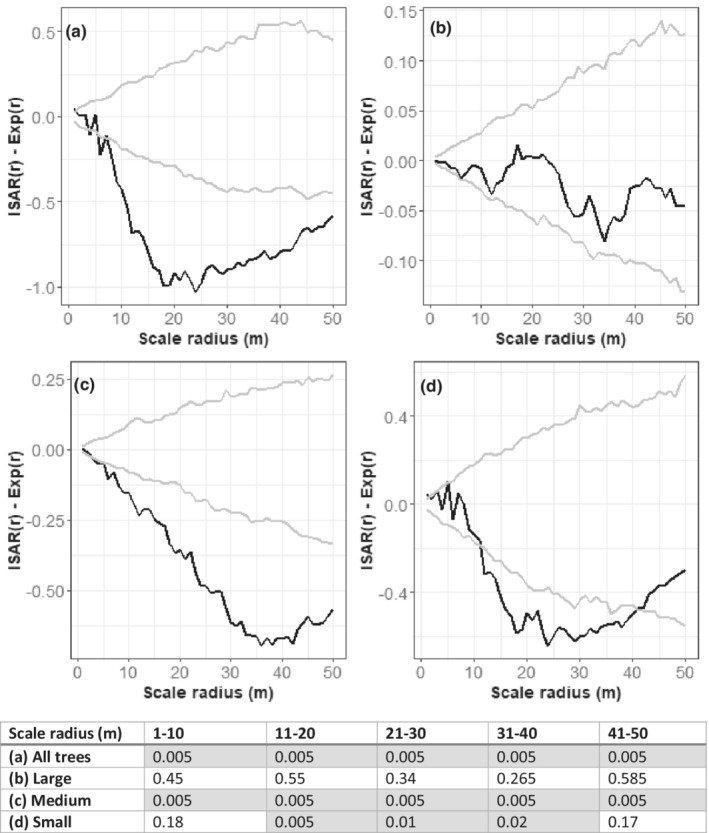
Results of ISAR analyses for *Cardwellia sublimis* (repeller). Black lines show ISAR observed – ISAR expected, which represents the observed ISAR function minus the expectation under a heterogenous Poisson null model. Gray lines represent the fifth highest and fifth lowest values of the 199 simulations of the null model. ISAR results are shown for (a) all individuals, (b) large individuals (≥70 cm dbh), (c) mid‐sized individuals only (≥30 and <70 cm dbh), and (d) small individuals only (<30 cm dbh). The table below gives the significance level (*p*) of the MAD goodness of fit test at increments of 10 m. Shaded cells highlight a result indicating a significant departure of the observed ISAR(r) from the null model ISAR at that particular increment

**FIGURE A12:**
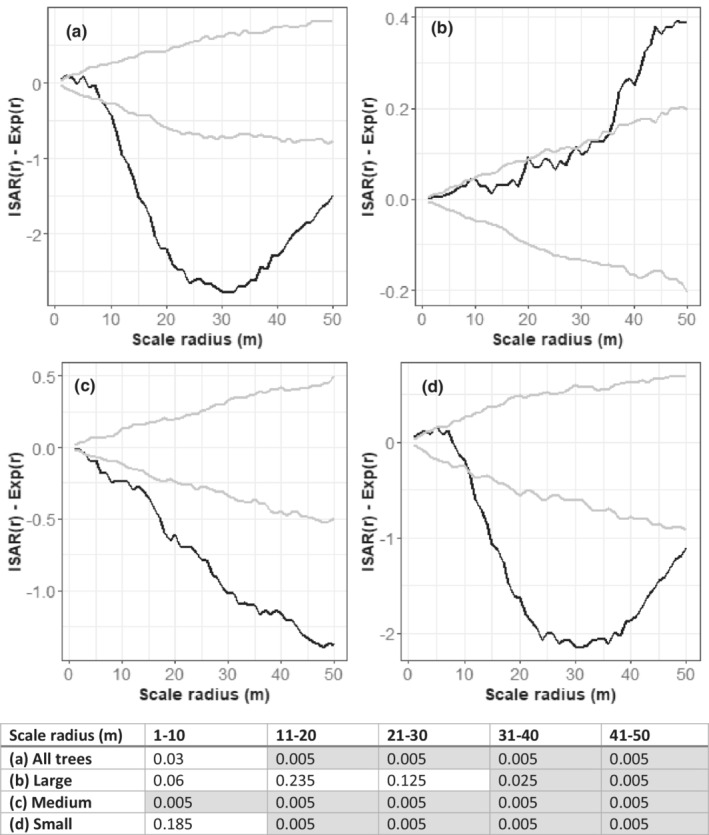
Results of ISAR analyses for *Darlingia darlingiana* (repeller). Black lines show ISAR observed – ISAR expected, which represents the observed ISAR function minus the expectation under a heterogenous Poisson null model. Gray lines represent the fifth highest and fifth lowest values of the 199 simulations of the null model. ISAR results are shown for (a) all individuals, (b) large individuals (≥70 cm dbh), (c) mid‐sized individuals only (≥30 and <70 cm dbh), and (d) small individuals only (<30 cm dbh). The table below gives the significance level (*p*) of the MAD goodness of fit test at increments of 10 m. Shaded cells highlight a result indicating a significant departure of the observed ISAR(r) from the null model ISAR at that particular increment

**FIGURE A13:**
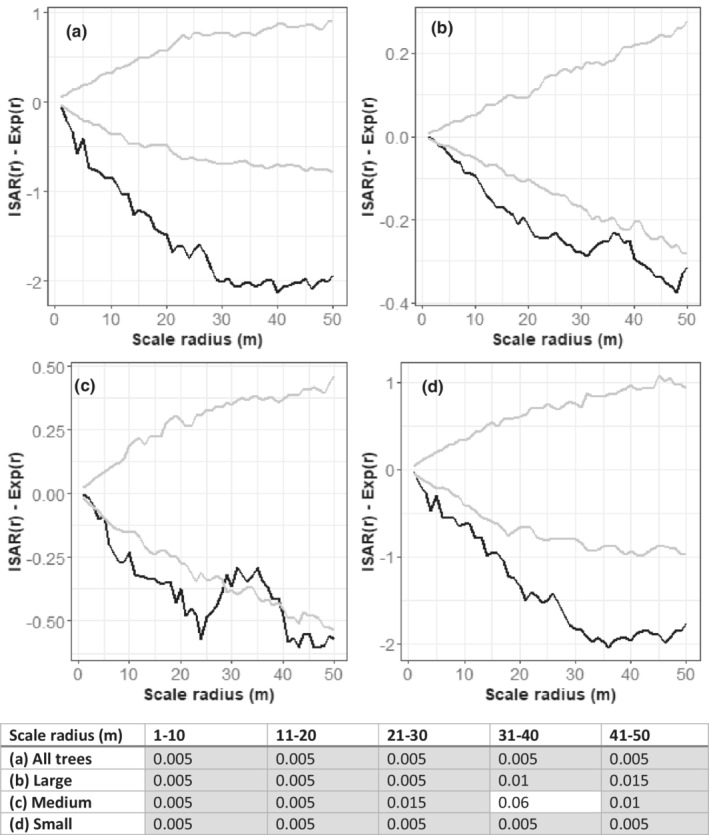
Results of ISAR analyses for *Endiandra monothyra* (repeller). Black lines show ISAR observed – ISAR expected, which represents the observed ISAR function minus the expectation under a heterogenous Poisson null model. Gray lines represent the fifth highest and fifth lowest values of the 199 simulations of the null model. ISAR results are shown for (a) all individuals, (b) large individuals (≥70 cm dbh), (c) mid‐sized individuals only (≥30 and <70 cm dbh), and (d) small individuals only (< 30 cm dbh). The table below gives the significance level (*p*) of the MAD goodness of fit test at increments of 10 m. Shaded cells highlight a result indicating a significant departure of the observed ISAR(r) from the null model ISAR at that particular increment

**FIGURE A14:**
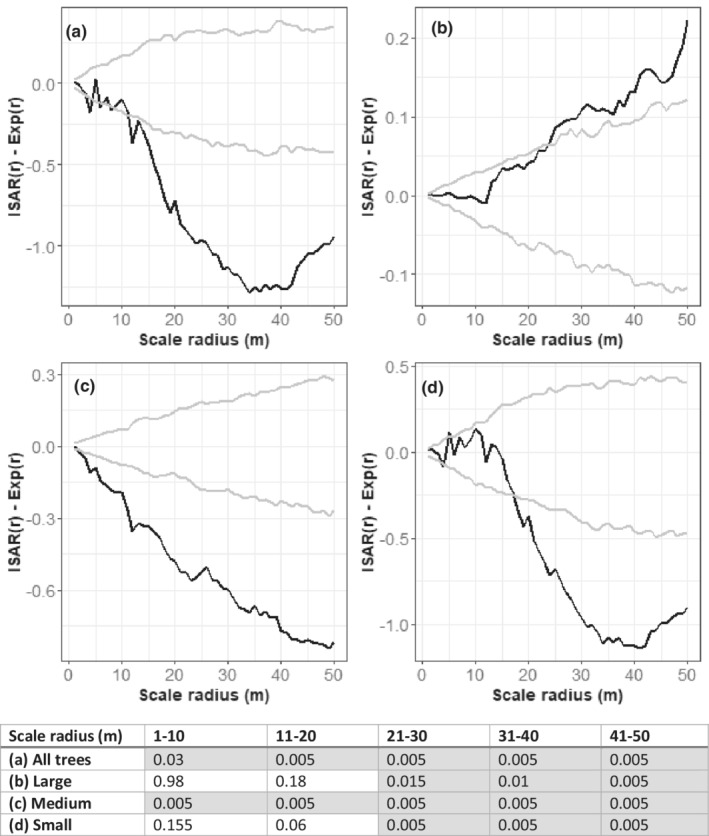
Results of ISAR analyses for *Litsea leefeana* (repeller). Black lines show ISAR observed – ISAR expected, which represents the observed ISAR function minus the expectation under a heterogenous Poisson null model. Gray lines represent the fifth highest and fifth lowest values of the 199 simulations of the null model. ISAR results are shown for (a) all individuals, (b) large individuals (≥70 cm dbh), (c) mid‐sized individuals only (≥30 cm and <70 cm dbh), and (d) small individuals only (<30 cm dbh). The table below gives the significance level (*p*) of the MAD goodness of fit test at increments of 10 m. Shaded cells highlight a result indicating a significant departure of the observed ISAR(r) from the null model ISAR at that particular increment

Examination of the detailed species results shows that the same 14 species consistently accumulate (seven species) (Figures A1, A2, A3, A4, A5, A6, A7) or repel (seven species) (Figures A8, A9, A10, A11, A12, A13, A14) diversity across most of the 50 m scale of the analysis (Table 2). The largest effects of accumulator species were on small individuals. Accumulator effects on big individuals were mostly neutral (Figures A1, A2, A3, A4, A5, A6, A7); however, two accumulator species significantly repelled big trees across most scales (Figures A4 and A7). Similarly, the largest effects of repeller species were on small and mid‐sized trees (Figures A8, A9, A10, A11, A12, A13, A14). Four of the seven repeller species significantly accumulated big trees particularly beyond the local neighborhood scale (beyond ~20 m) (Figures A9, A10, A12, A14), and another species had neutral effects on big individuals (Figure A11).

**TABLE 2:** Characteristics of consistent accumulator and repeller species

Species	Abb.	ISAR type	Fruit	Shade tolerance	Layer	# of stems			
						Total	small	mid	big
*Apodytes brachystylis*	Apo_bra	accum	fleshy	shade tolerant	sub canopy	279	278	1	0
*Citronella smythii*	Cit_smy	accum	fleshy	shade tolerant	sub canopy	177	174	3	
*Franciscodendron laurifolium*	Fra_lau	accum	woody	light demanding	canopy	352	320	32	
*Gillbeea adenopetala*	Gil_ade	accum	woody	moderate	canopy	316	275	40	1
*Levieria acuminata*	Lev_acu	accum	fleshy	shade tolerant	sub canopy	173	172	1	
*Medicosma fareana*	Med_far	accum	fleshy	shade tolerant	sub canopy	276	275	1	
*Symplocos paucistaminea*	Sym_pau	accum	fleshy	shade tolerant	sub canopy	105	105		
*Acronychia vestita*	Acr_ves	repel	fleshy	moderate	sub canopy	234	222	12	
*Alphitonia whitei*	Alp_whi	repel	fleshy	light demanding	canopy	851	813	38	
*Alstonia muelleriana*	Als_mue	repel	woody	light demanding	canopy	434	363	68	3
*Cardwellia sublimis*	Car_sub	repel	woody	light demanding	canopy	1541	1239	269	33
*Darlingia darlingiana*	Dar_dar	repel	woody	light demanding	canopy	688	299	87	2
*Endiandra monothyra*	End_mon	repel	fleshy	moderate	canopy	445	418	26	1
*Litsea leefeana*	Lit_lee	repel	fleshy	light demanding	canopy	1837	1676	161	

**FIGURE 5:**
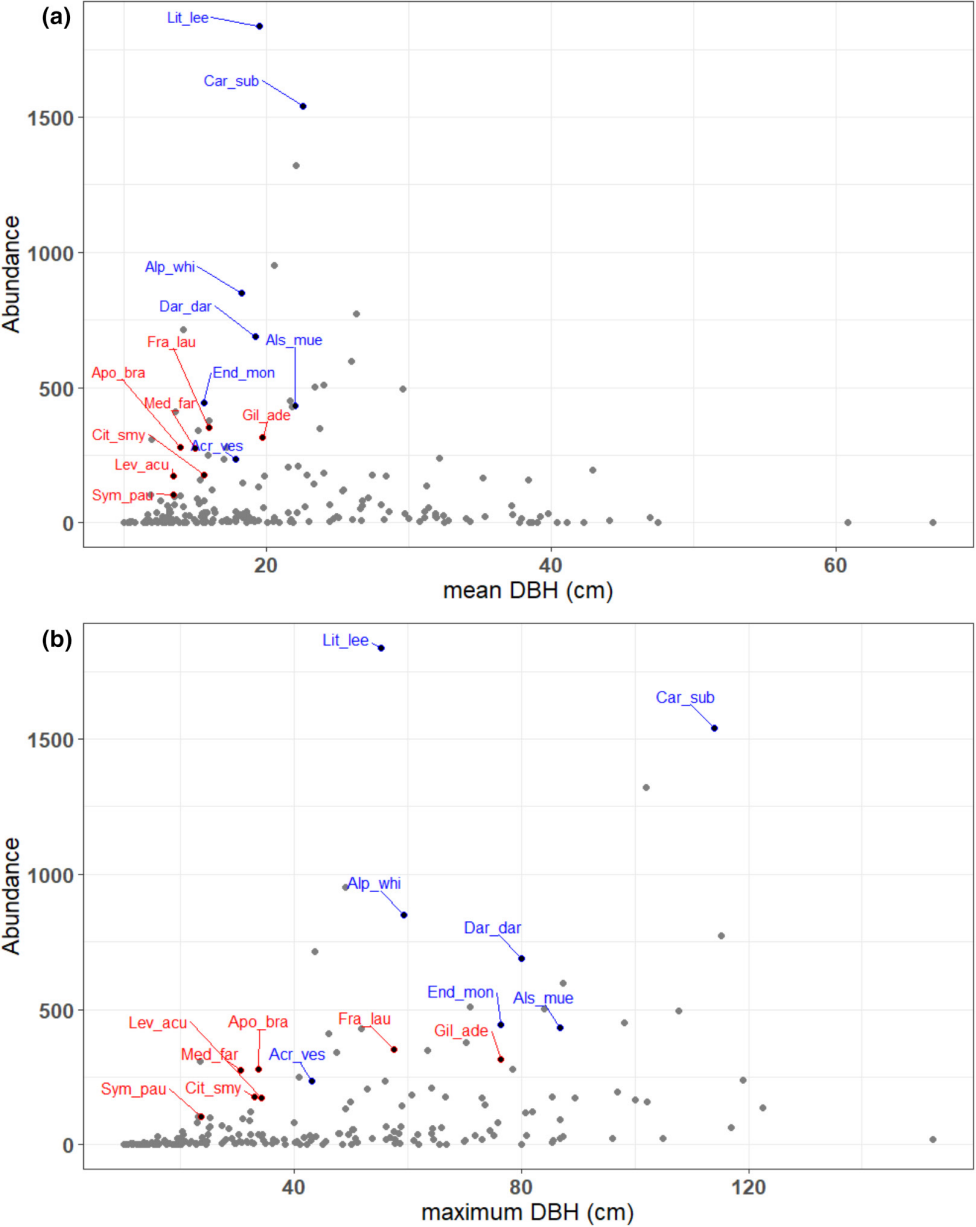
Abundance and (a) mean dbh and (b) maximum dbh for all species (gray circles) highlighting repeller species (blue text) and accumulator species (red text). Species abbreviations are given in Table 2.

**FIGURE 6:**
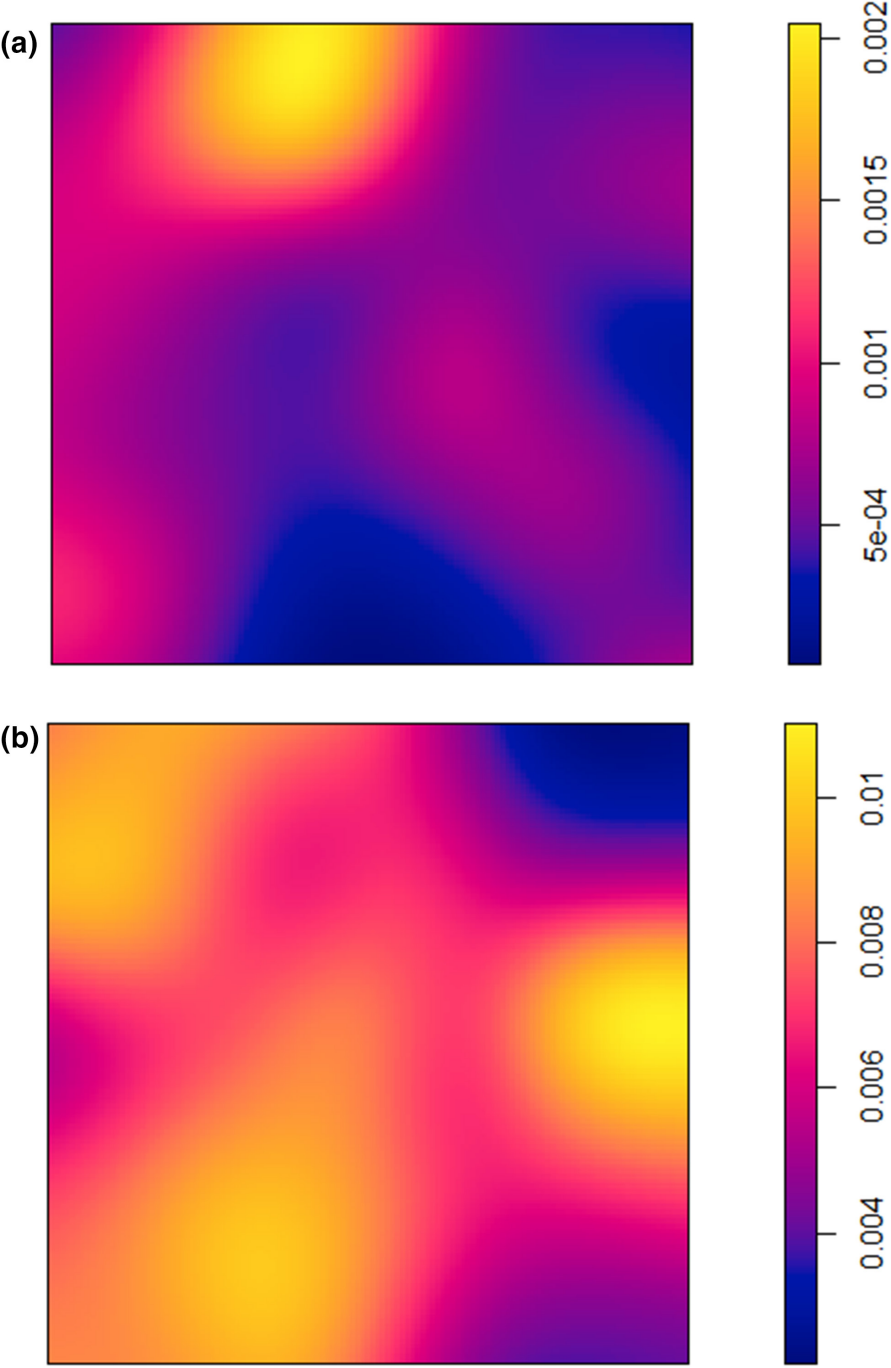
Density of (a) accumulators and (b) repellers across the plot

Repeller species tended to have higher abundance and be bigger on average than accumulator species (Figure 5). Accumulator species were more often shade‐tolerant, sub‐canopy species compared with repeller species, which tended to be light demanding or moderately light‐demanding canopy species (Table 2). Five of the seven accumulators and four of the seven repeller species were fleshy‐fruited (primarily bird‐dispersed). The density of accumulator species and repeller species was largely offset across the plot (Figure 6).

## 4 DISCUSSION

Our analysis suggests that big individuals exert some spatial structure on mid‐size and other big trees with lower than expected species richness across the 25 ha plot at relatively close distances (up to 27 m). This result is not surprising considering that the physical space big trees occupy leaves little additional room for other mid or big trees in close proximity. The effect of big trees on spatial structuring of small trees is neutral.

Up to 50% of abundant species (*n* = 53) display patterns of non‐random repelling of other species in local neighborhoods (<5 m), declining to between 10 and 20% across larger neighborhood sizes (~ >7 m). A much smaller proportion of species (<10%) display patterns of accumulation in local neighborhoods though this proportion rises at larger neighborhood sizes to approximately the same proportion as for repellers (i.e., 10–20%). Contrasting patterns have been indicated in other tropical forests with most displaying a predominance of accumulators at local neighborhood scales (Chanthorn et al., 2018; Nguyen et al., 2018; Tsai et al., 2015; Wiegand et al., 2007), while only a few showed a predominance of repellers (Fibich et al., 2021; Wiegand et al., 2007).

Rarely have consistent species‐specific signatures on spatial diversity structures been demonstrated beyond local neighborhoods (Chanthorn et al., 2018; Punchi‐Manage et al., 2015; Wiegand et al., 2007). At Robson Creek, 14 species (seven repellers and seven accumulators) consistently influence species richness across neighborhoods up to ~40 m radius (Table 2). The species that we identify as consistent repellers repel species across most of the analysis area (Table S1), whereas the consistent accumulators are mostly neutral at distances from about 1–10 m, then accumulate consistently across the remainder of the radii. Only one of the abundant species at Robson Creek, *Levieria acuminata*, shifted from being a significant repeller at short distances (<10 m) to significantly accumulating at larger distances (11–50 m). *Levieria* tends to colonize shaded alluvial areas such as old creek lines. As a result, it is often abundant where it occurs at a local scale but is uncommon in other habitats (i.e., beyond the local neighborhood).

The patterns we observe suggest multiple mechanisms are responsible for structuring species diversity around focal species. A primary mechanism appears to be differences among species in resource partitioning related to disturbance‐induced variability in light, which is manifested in spatial heterogeneity in species distributions across the plot. Our accumulators were mostly shade‐tolerant (except *Franciscodendron laurifolium*) suggesting they occupy a more stable (less frequently disturbed) space in the landscape where a diversity of other shade‐tolerant species may co‐occur. Shade‐tolerant species comprise 61% of species (>10 cm dbh) at the plot, with moderately shade tolerant species comprising an additional 27% of species. As such, the potential for higher diversity in more stable, shaded areas is elevated. In contrast, there were no shade‐tolerant species among the repellers. Light‐demanding species comprise only 12% of the species in the plot making these results particularly significant. We suggest that disturbance events allow a small number of light‐demanding species to colonize canopy gaps en masse, effectively excluding other species and lowering diversity in those areas, resulting in the repeller pattern. This is borne out by the fact that repeller density is highest (Figure 6b) in areas where small tree richness is relatively low (Figure 3b). In contrast, the pattern of accumulation around the light‐demanding *F. laurifolium* suggests positive inter‐specific interactions or intra‐specific competition leading to a lower density of conspecifics and thus higher richness of heterospecifics (e.g., Janzen–Connell effects).

Only two of the seven repeller species (*Acronychia vestita* and *Endiandra monothyra*) maintained their repeller effect on big trees across scales, and four of our repeller species tended to show accumulator effects with big individuals at larger scales. However, no accumulator species maintained an accumulator effect on big individuals. Most of our repeller and accumulator individuals fall into the small tree category themselves (88% and 95% of individuals respectively) and it is not surprising that big, old individuals are little influenced by negative interactions with them. The accumulator pattern shown by some repeller species on big individuals at larger scales suggests positive interactions. This can be seen in the area representing an overlap between a high richness of big trees (Figure 3f) and a high density of repeller individuals (Figure 6b). Big, old individuals may persist as legacies of past conditions in areas that are subsequently disturbed, they may be more resilient to events that cause minor disturbances (e.g., storms), or they may actually generate canopy gaps as they lose branches or limbs allowing light‐demanding species to recruit in their neighborhood.

We found that repellers tended to have higher abundance than accumulators. The two most abundant species on the plot were repellers (*Litsea leefeana n* = 1837 and *Cardwellia sublimis n* = 1541). Repeller species comprise 26% of the total number of individuals in the plot, whereas accumulators comprise 7% of total individuals. In addition, repellers tended to be bigger (in dbh) on average, reach greater maximum diameter than accumulators (Figure 5), and were more often canopy species (Table 1). This finding is in contrast to results from a 25 ha subtropical plot in Taiwan where it was found that species with relatively high abundance ranks and larger size tended to accumulate. The authors suggested that abundance interactions act to determine the neighborhood species richness of plant communities (Tsai et al., 2015). Fibich et al. (2021) also found that abundant species were often accumulators. This effect was thought to be driven by density‐dependent mortality factors that promote the establishment of heterospecific seedlings near the adult plants of dominant species (i.e., per Janzen–Connell patterns, Janzen (1970)). Given the light‐demanding nature of the repeller species at Robson Creek, and that they are abundant across much of the plot, we suggest that opportunities for a large diversity of shade‐tolerant species to establish in their neighborhood are limited by competition for space and undesirable disturbance regimes.

It is possible that seedling and sapling richness and abundance in disturbed areas, which we have not captured here, are relatively high as expected under theories of gap phase dynamics in tropical forests (Hubbell et al., 1999) and that we would see some of our repellers switch to accumulators at local scales if individuals <10 cm dbh were included. For example, Fibich et al. (2021) analyzed spatial diversity patterns in tropical forest plots for all trees >1 cm dbh and found much higher proportions of accumulators (72%–78%) when including all species. This pattern was driven by small trees (<10 cm dbh); when considering trees >10 cm dbh, the proportion of accumulators ranged from 7% to 29%, and the proportion of accumulators and repellers in local neighborhoods was similar to our results. Repellers peaked at ~3 m and accumulators at ~12 m (Fibich et al., 2021).

Punchi‐Manage et al. (2015) and Chanthorn et al. (2018) documented stronger accumulator effects for species with animal dispersed seeds. Tropical avian frugivores are known to disperse many species of plants and spatially contagious, heterospecific fleshy‐seed dispersal has been demonstrated in several tropical locations (Clark et al., 2004; Wright et al., 2016). Five of seven of our accumulators were animal dispersed; however, four of the seven repeller species also had fleshy fruits. Given that 80% of species (168 of 207) at Robson Creek are fleshy fruited, this result reflects a surprising proportion of woody fruited species among repellers. Among all light‐demanding species on the plot, 40% have woody fruits (compared with only 13% of shade‐tolerant species), which helps explain the high proportion of woody fruited repellers, which are also all light‐demanding. Wind‐dispersed, light‐demanding species may be more likely to repel other species due to creation of a large seed bank in the local neighborhood and rapid recruitment after disturbance.

Many tropical forests are undergoing changes in disturbance regimes as a result of human activity (Lewis et al., 2015; McDowell et al., 2020). There is strong evidence that higher levels of species diversity confer resilience on tropical forests and facilitate faster recovery times to disturbance (Adolf et al., 2020; Schmitt et al., 2020). We have previously described how Australian rainforests have a relatively high proportion and diversity of species that are capable of reaching large size, potentially affording greater resilience to disturbance in terms of recovery of biomass (Bradford & Murphy, 2019). However, an increase in the frequency or intensity of disturbances, for example, from increased drought or storm events, has the potential to favor woody fruited, repeller species resulting in long‐term declines in species diversity, and potentially compromising resilience to future disturbance regimes.

## 5 CONCLUSIONS

In this study, we have demonstrated that big trees exert limited spatial structure on most other individuals (small trees) but tend to have lower than expected species diversity of mid‐sized and other big trees in their neighborhood (up to ~30 m). Most abundant species in this diverse tropical forest also leave no strong spatial signature on tree species diversity in their surrounding neighborhood. However, a small number of abundant species leave a consistent spatial fingerprint of higher or lower than expected tree species diversity across scales of up to 50 m radius. We suggest that niche partitioning along disturbance gradients is a primary mechanism driving spatial richness patterns associated with accumulator and repeller species. Niche differentiation as a driver of species spatial richness structures has been shown to be pervasive in heterogeneous and diverse tropical forests (Brown et al., 2013; Tsai et al., 2015).

The repeller species *L. leefeana*, *C. sublimis*, *Alphitonia whitei*, and *Darlingia darlingiana* partly fit the definition of “foundation species” in the Robson Creek plot. They are large, abundant, and lower species diversity in their local neighborhood (Ellison et al., 2019). Further exploration of spatial relationships between these candidate foundation species and co‐occurring species (e.g., their influence on betadiversity) will provide important insights into their role in structuring diversity across landscapes.

## AUTHOR CONTRIBUTIONS

**Helen Murphy:** Conceptualization (lead); data curation (supporting); formal analysis (lead); funding acquisition (equal); investigation (equal); methodology (equal); writing – original draft (lead). **Matt G Bradford:** Conceptualization (supporting); data curation (lead); formal analysis (supporting); funding acquisition (equal); investigation (equal); methodology (equal); writing – original draft (supporting); writing – review and editing (supporting).

## CONFLICT OF INTEREST

The authors declare no competing financial or personal interests.

## Data Availability

Full vegetation survey data for the Robson Creek 25 ha plot including species, diameter at breast height (dbh), tree height and geographic coordinates for all trees ≥10 cm dbh (Bradford, 2018): TERN Ecosystem Research Infrastructure Data Discovery Portal. https://portal.tern.org.au/vegetation‐data‐direct‐plot‐2015/21218
